# Factors that influence the uptake of postnatal care among adolescent girls: a qualitative evidence synthesis

**DOI:** 10.1136/bmjgh-2022-011560

**Published:** 2023-05-03

**Authors:** Dena Javadi, Emma Sacks, Vanessa Brizuela, Kenneth Finlayson, Nicola Crossland, Etienne V Langlois, Daniela Ziegler, Venkatraman Chandra-Mouli, Mercedes Bonet

**Affiliations:** 1 Social and Behavioral Sciences, Harvard University T.H. Chan School of Public Health, Boston, Massachusetts, USA; 2 Department of International Health, Johns Hopkins University, Baltimore, Maryland, USA; 3 UNDP/UNFPA/UNICEF/WHO/World Bank Special Programme of Research, Development and Research Training in Human Reproduction (HRP), Department of Sexual and Reproductive Health and Research, World Health Organization, Geneva, Switzerland; 4 Research in Childbirth and Health (ReaCH) Unit, School of Community Health and Midwifery, University of Central Lancashire, Preston, UK; 5 Partnership for Maternal, Newborn & Child Health (PMNCH), World Health Organization, Geneva, Switzerland; 6 Library, Centre Hospitalier de l'Universite de Montreal, Montreal, Québec, Canada

**Keywords:** review, maternal health, qualitative study, health systems, public health

## Abstract

**Background:**

Adolescent pregnancy is associated with increased risk of maternal and child morbidity and mortality globally. Access to safe, appropriate and affordable antenatal, childbirth and postnatal care (PNC) is essential in mitigating this risk. PNC is an often undervalued, underused, and understudied component of the continuum of maternal health services; however, it provides an important opportunity for adolescent girls to have access to health information and resources as they navigate the transition to motherhood and/or recovery from childbirth. This qualitative evidence synthesis seeks to highlight the experiences and perspectives of adolescent girls and their partners in accessing and using routine PNC.

**Methods:**

Papers were selected from a primary review on PNC where a global search of databases was conducted to identify studies with qualitative data focused on PNC utilisation. Within this primary review, a subset of studies focused on adolescents was tagged for subanalysis. A data extraction form drawing on an a priori framework was used to extract data from each study. Review findings were grouped across studies and mapped onto relevant themes, which were then adapted, as appropriate, to best reflect emergent themes from included studies.

**Results:**

Of 662 papers identified for full text review, 15 were included in this review on adolescents’ experiences. Fourteen review findings were mapped onto four themes including: resources and access, social norms, experiences of care, and tailored support needs.

**Conclusion:**

Improving uptake of PNC by adolescent girls requires multipronged approaches in improving availability of and access to adolescent-sensitive maternal health services and reducing feelings of shame and stigma in the postpartum period. Much should be done to address structural barriers to access, but tangible steps to improving the quality and responsiveness of available services can be taken immediately.

**PROSPERO registration number:**

CRD42019139183.

**WHAT IS ALREADY KNOWN ON THIS TOPIC**

- Improved access to high-quality, appropriate, and affordable maternal and neonatal health services is critical in addressing the challenges associated with complications during and after pregnancy among adolescents and their infants.
- Structural barriers to access, such as financial constraints and lack of information, as well as sociocultural barriers, such as perceived stigma associated with adolescent pregnancy, negatively impact uptake of maternal health services, including postnatal care (PNC), which is in turn associated with poor maternal and neonatal health outcomes.

**WHAT THIS STUDY ADDS**

- This study emphasises the perspectives of adolescent girls and highlights the unique challenges and barriers they face in seeking and benefitting from routine PNC.
- To enhance adolescents’ uptake of PNC, services should be tailored (including health promotion services) to respond to adolescents’ and young people’s needs in a judgment-free and stigma-free environment.

**HOW THIS STUDY MIGHT AFFECT RESEARCH, PRACTICE OR POLICY**

- Addressing health workforce challenges, such as burnout and staffing shortages, is necessary in enabling health providers to deliver high-quality PNC.
- Health providers should have enhanced training and ongoing support in responding to the specific needs and preferences of adolescents during the postnatal period in order to reduce stigma, and to create a safer space for adolescents to voice concerns and questions.
- PNC should more actively and meaningfully engage adolescent girls’ partners and families, where appropriate, and leverage peer-to-peer support networks to facilitate the transition to parenthood and/or recovery from childbirth.
- More research and policy are required to support the implementation of effective adolescent-sensitive interventions.

## Introduction

The WHO defines adolescents as individuals aged 10–19 years.^1^ Adolescent pregnancy is associated with increased risk of maternal and child morbidity and mortality globally.^2 3^ The first 6 weeks (42 days) after birth, the postnatal period, are critical for both women and newborns as a majority of maternal and neonatal health (MNH) complications and deaths occur during this period.^4 5^ Equitable access to and quality of clinical care—in addition to addressing structural determinants such as reproductive rights, gender equity, education, economic power and racial justice—could mitigate these risks.^6–9^

Globally, adolescents have significantly lower coverage of PNC as compared with their older counterparts.^10^ While research has explored trends related to adolescent pregnancy,^11 12^ its drivers,^9 12^ complications,^13–15^ prevention,^16^ use of antenatal care (ANC),^17^ and responses to complications in the postnatal period,^18^ evidence is limited on the factors related to accessibility and use of postnatal care (PNC) services following pregnancies among adolescents. Additionally, while structural determinants are explored in designing policies and programmes to reduce the number of unintended pregnancies,^17^ less is known around addressing barriers in the access to and use of PNC by adolescent girls, particularly regarding the design and implementation of appropriate, acceptable and adolescent-centred care.^19^

In a review of the quantitative evidence on use of maternal health services by adolescents, access to and use of ANC services, and presence of skilled birth attendants (SBA) have been identified as predictors of use of PNC services.^20^ Furthermore, wealth quintiles, mother’s education, partner’s education, employment status, media exposure, urban residence, mass media exposure, and religion were identified across studies as contributing to access to maternal health services.^20 21^ Little qualitative evidence is available regarding the appropriateness and quality of existing PNC, the opinions of adolescents regarding PNC or the specific PNC needs of the adolescent age group given social and environmental factors including interrupted education, uncertain housing status, limited and unreliable incomes, weak family support, and lack of decision-making support. Where some evidence exists on quality of and satisfaction with PNC, mothers who are not adolescents tend to report higher quality scores.^22^ While adolescent girls may express similar desires identified among all women for the postnatal period—for example, enhancing self-esteem, competence and autonomy, and adapting to new familial and social roles—they may require different approaches during PNC to meet their unique needs and priorities.^23^ Therefore, it is important to synthesise existing evidence specifically on adolescent girls’ perceptions of and experiences with PNC, their wants and needs, and how these contribute to the use of and satisfaction with services. This qualitative evidence synthesis (QES) seeks to address this knowledge gap by highlighting key issues raised by adolescent girls and their partners in accessing and using PNC.

Using a subset of data from a larger QES on the views and perspectives of postnatal women and the factors that influence uptake of PNC services as the source QES,^24^ this subanalysis synthesises the qualitative evidence specific to the subpopulation of adolescent girls. The aim is to summarise factors that influence the uptake of routine PNC from the perspective of adolescent girls. Better understanding of adolescent girls’ perspectives can improve the development of adolescent-sensitive MNH policies, including PNC, not least through meaningful adolescent engagement. This can both enhance the quality of routine PNC made available to adolescents and inform targeted programming to address any specific challenges they may face in accessing and using PNC.

## Methods

We conducted a QES using a framework approach and thematic techniques to analyse and descriptively synthesise relevant qualitative data.^25^ Descriptive themes were generated and organised into review findings that were assessed for confidence using the GRADE (‘Grading of Recommendations, Assessment, Development and Evaluations’)-CERQual (‘Confidence in the Evidence from Reviews of Qualitative research’) tool.^26^ This review uses the subset focused on adolescent girls’ and their partners’ views and experiences of PNC to identify themes that are prominent in this subpopulation.

The search strategy and study selection methods of the primary QES are summarised elsewhere.^24^ The QES included qualitative or mixed-methods studies that included a qualitative component in the study design (eg, ethnography), qualitative data collection (eg, interviews, focus groups) or method of analysis (eg, thematic analysis). Databases included MEDLINE (OVID), PubMed, CINAHL (EBSCO), EMBASE (OVID), EBM-Review (OVID) and a grey literature search via BASE (Bielefeld University Library), OpenGrey and the WHO website. We used a comprehensive search strategy to maximise data retrieval and, depending on database functionality, used a broad range of qualitative descriptors (ethnography, phenomenology, grounded theory, focus group, interview, etc) to optimise the identification of qualitative research studies. Our aim with the primary search was to cast the net as wide as possible to try and incorporate a range of views from different settings and contexts in accordance with the global nature of the review. The search strategy covered papers published from inception to December 2019. There were no language restrictions. Duplicates were removed through the EndNote X9 software using a method developed by Bramer *et al*.^27^

Records were collated into Covidence software where duplicates were removed, and records were screened based on title and abstract. Members of the team (ES, MB, VB) independently screened titles and abstracts against the inclusion/exclusion criteria and flagged studies that both met the general inclusion criteria and were specific to and tagged as views of adolescent girls and their partners. We did not define an age cut-off for adolescents, but followed the definitions used by the study authors; studies in which the population was defined as ‘adolescent’, ‘teenage’ or ‘young’ were included. Inclusion and exclusion criteria for the overall study and the specific analysis can be found in table 1. As the aim of the QES was to describe factors related to routine PNC for adolescent girls, studies focusing only on specialised services for known conditions or high-risk groups were not included.

**Table 1:** Inclusion and exclusion criteria

Inclusion criteria for primary review	Exclusion criteria for primary review	Additional inclusion/exclusion criteria for the current analysis on adolescent girls’ and adolescent partners’ views on routine postnatal care
Studies including women and/or their partners/families who were considered to be healthy in the postnatal period, and/or who had a healthy newborn Studies where at least some of the extractable data were women’s, and/or their partners/families, own accounts of their views and experiences of the nature of, provision of and/or seeking of postnatal care after birth, irrespective of parity, mode of birth or place of birth Studies involving postnatal care experiences with or without interaction with the health system but relating to health care (home-based, community-based care, etc.) Studies from high-income, middle-income and low-income countries	Studies reporting on views/experiences of, or access to, maternity services generally with no specific data on postnatal care. Women with known complications/health conditions (eg, depression), or after severe morbidity (eg, near-miss) Services for specific conditions (eg, HIV), or high-risk populations (eg, multiples, preterm, low birth weight, malformations). Specific interventions for a singular condition (eg, breastfeeding support, family planning, mental health) or postnatal education only (eg, parenting education). Studies related to care of postnatal complications or intensive care for women or newborns. Mixed-methods studies reporting qualitative data without using a recognised qualitative approach to analysis. Case studies, conference abstracts and unpublished PhD or Master’s theses. Systematic reviews (although reference lists were reviewed).	Inclusion Studies including adolescent girls’ (or partners’) accounts. Studies focused on adolescent girls’ (or their partners’ views where available) and experiences of postnatal care. Quality appraisal score of A–C Exclusion Studies focused on postnatal experiences of groups other than adolescent girls

### Data extraction and analysis

Data extraction, analysis and quality appraisal proceeded concurrently and broadly followed the ‘best fit’ framework approach described by Carroll *et al,*^28^ incorporating thematic synthesis techniques^25^ to develop new themes where emerging data failed to fit our a priori framework. Quality appraisal was conducted using an instrument developed by Walsh and Downe,^29^ and modified by Downe *et al*,^30^ with studies rated against 11 predefined criteria and receiving a score of A–D representing credibility, transferability, dependability, and confirmability of results. Studies scoring C or higher were included in the analysis.

Based on previous related reviews of ANC^31^ and intrapartum care,^23^ we used a deductive approach to develop a thematic framework comprising four broad concepts (resources and access; behaviours and attitudes; external influences; what adolescent girls want and need) as well as a number of subthemes. Data extraction was done using a form developed for this review in Microsoft Excel to record study details, themes identified by authors, their alignment with the a priori framework, and supportive quotes (see online supplemental appendix 1 for the data extraction form). Differing perspectives from study team members on identified themes were settled through discussion and consensus building. Two studies in Portuguese were reviewed by one of the review authors fluent in the language and extracted data were translated to English.

10.1136/bmjgh-2022-011560.supp1 Supplementary data

On confirming themes and subthemes identified through the review, the study team applied the GRADE-CERQual tool^26^ to assess the confidence of each finding based on methodological limitations, relevance, coherence and having sufficient data to support findings. Each finding was graded as ‘high’, ‘moderate’, ‘low’ or ‘very low’. The grading was agreed by consensus between two study team members (DJ, ES). Any discrepancies in grading were resolved by a third review author (VB). QES findings were then grouped into higher order analytical themes agreed on by all authors.

## Results

**Figure 1:**
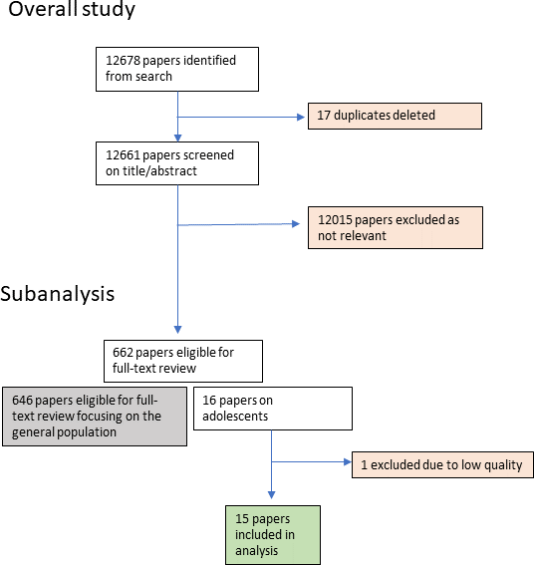
Flow diagram of included studies for subanalysis on adolescent girls.

The general review yielded 12 678 papers, with 17 duplicates deleted and 12 015 excluded.^24^ Title and abstract screening identified 16 studies as being focused on the experiences of adolescent girls in accessing PNC, 15 were included in full-text data extraction with one being excluded due to not meeting the eligibility criteria (see figure 1). Of these, four represented upper middle-income countries, two represented lower middle-income countries and one represented a low-income country while eight represented high-income countries. Countries included Brazil (four studies),^32–35^ Canada,^36^ Indonesia,^37^ Eswatini (formerly known as Swaziland),^38^ Uganda,^39^ the UK (four studies),^40–43^ and the USA (three studies).^44–46^ Thirteen of the 15 studies included adolescent girls (with 2 also including young women up to age 24 years) as participants while 2 included both adolescent girls and their partners. All studies were qualitative in nature with one incorporating mixed methods. Study characteristics are summarised in table 2.

**Table 2:** Study characteristics

Study	Country (income level)	Context	Study design	Participants
Atuyambe *et al*^39^	Uganda (low)	Rural, health facility	Qualitative using grounded theory, using FGD and KII	Adolescent girls (aged 16–19 years) and midwives in charge of maternity units
Bergamaschi and Praça^32^	Brazil (upper middle)	Urban, health facility, home	Qualitative using CSS*	Primiparous adolescent girls
Dumas *et al*^46^	USA (high)	Urban, health facility	Qualitative using FGDs	Adolescent girls (aged 18–24 years) (first child in past 5 years)
Erfina *et al*^37^	Indonesia (lower middle)	Urban, health facility	Descriptive phenomenology† using interviews	Primiparous adolescent girls (aged 16–19 years)
da Silva *et al*^33^	Brazil (upper middle)	Urban, health facility	Qualitative thematic analysis using semi-structured interviews	Adolescent girls (aged 10–19 years) receiving care at study hospital
de Melo *et al*^35^	Brazil (upper middle)	Urban, home, health facility	Qualitative thematic analysis using in-depth interviews	Adolescent girls 20 days post partum
Hunter *et al*^43^	UK (high)	Urban/rural, community-based	Qualitive using constructivism‡ and FGDs, parent group sessions	Adolescent girls (aged 16–20 years) attending group sessions
Mngadi *et al*^38^	Eswatini (Swaziland) (lower middle)	Urban, health facility	Exploratory using in-depth interviews	Adolescent girls aged 10–19 years
Muzik *et al*^44^	USA (high)	Urban, community-based	Community-based participatory research using FGDs and in-depth interviews	Black or ethnic minority teenage girls (aged 13–20 years) or women who had been a teenage mother in the past 10 years
Peterson *et al*^36^	Canada (high)	Urban, health facility	Phenomenology using FGDs and interviews	Unmarried mothers up to 19 years of age
Recto and Champion^45^	USA (high)	Urban, school/community-based	Descriptive using interviews	Mexican-American adolescent girls aged 15–19 years
Robb *et al*^42^	UK (high)	Urban, health facility	Phenomenological approach	Young women who had children between the ages of 2 and 13 months and were on the caseload of health visitors
Ross *et al*^41^	UK (high)	Urban, community	Qualitative using interviews	Young parents aged 16–19 years (girls), 15–25 years (boys and men)
Smith and Roberts^40^	UK (high)	Urban, health facility	Mixed methods using surveys and FGDs	Young parents aged 15–25 years
Vieira *et al*^34^	Brazil (upper middle)	Urban, health facility	Personal testimony (semi-structured interviews)	Primiparous women aged 10–19 years, who resided in the city

*CSS (collective subject’s speech): consists of ‘reading the speeches of each interview and the consequent identification of key expressions—significant extracts for the study—which originate the main ideas present in each of the individual speeches. Afterwards, these were gathered by content affinity in a synthesis that originated the CSS, identified by themes.”^32^

†Phenomenology is the study of experiences and ‘has transitioned from descriptive phenomenology, which emphasises the “pure” description of people’s experiences, to the “interpretation” of such experiences, as in hermeneutic phenomenology’.^65^

‡Constructivism is a learning theory that ‘recognises that the findings of qualitative research can be influenced by the world view of the researcher, who is intimately bound up in the process of data generation’.^43^

CSS, collective subject’s speech; FGD, focus group discussions; KII, key informant interviews.

The QES generated 14 review findings. These findings were mapped to the a priori framework and compared with the amended themes in the primary review.^24^ In the primary review, *resources and access* was split into *access and availability* and *physical and human resources. Behaviours and attitudes* was amended to *social norms* and *what women want and need* was changed to *experiences of care*.^24^ In this review focused on adolescent girls, some of the same amendments were appropriate, including changing *behaviours and attitudes* and *what adolescent girls want and need* to *social norms* and *experiences of care,* respectively. Other changes diverged from the primary review in order to more specifically represent adolescents’ perspective. *Resources and access* was kept as one category and combined with *external influences*. An additional theme, *tailored support needs*, was added based on findings across the included papers. This was an area that appeared to be of significant concern for adolescents, particularly in terms of feeling unprepared for the transition to parenthood and requiring extra information and different types of support during PNC—from family, partners, and/or peers—without judgement. While this new theme has some overlap with *social norms* and *experiences of care*, we felt a dedicated theme was necessary to emphasise the specific needs of this subpopulation. Table 3 highlights review findings mapped across the four themes along with the contributing papers, selected supporting quotes, and the GRADE-CERQual score.

**Table 3:** Themes and review findings

Theme	Review finding	Contributing papers	Supporting quote	GRADE-CERQual
Resources and access	*Access barriers*: access barriers included lack of health insurance, not having accessed ANC, cost and/or lack of transport to health facilities and direct costs associated with PNC services.	4 studies: Atuyambe *et al*^39^ (Uganda); Dumas *et al*^46^ (USA); da Silva *et al*^33^ (Brazil); Mngadi *et al*^38^ (Eswatini)	“I stopped using Medicaid at 19 and that was really hard, and I just went to the emergency room every time. For anything…I went almost every day”.^46^ (USA) “When I was pregnant, what prevented me from seeking health care was lack of transport money because my legs were a problem. I used to live far away in the hills and I could not ask anyone to take me on a bicycle because I would be asked for money. So I decided to rely on my grandmother’s traditional herbs”.^39^ (Uganda)	Moderate
	*Availability of desirable services*: lack of availability and/or information regarding services that adolescent girls would feel comfortable accessing affected both uptake of PNC and adolescent girls’ uncertainty with available services (sometimes resulting in reliance on more familiar practices by family or other community members).	4 studies: Muzik *et al*^44^ (USA); Recto and Champion^45^ (USA); Mngadi *et al*^38^ (Eswatini); Atuyambe *et al*^39^ (Uganda)	“For us the Baganda we undergo a traditional practice we call ‘OKUFUGIKA’. The practice will make the child who stays on earth to be a clever person. But if they [health workers] throw the ‘second child’ [placenta] into a latrine the child who stays on earth become dull and not intelligent”.^39^ (Uganda)	Moderate
	*Facility environments and resources*: the physical state and feel of health facilities and their available resources played an important role in adolescents’ desire to seek care at health facilities; cleanliness, availability of water, state of available bedding, odours and associations with illness influenced adolescents’ health-seeking behaviours.	2 studies: Atuyambe *et al*^39^ (Uganda); Hunter *et al*^43^ (UK)	“Although we have these water tanks here water is not accessible to patients. This water is rationed for conducting deliveries and cleaning the floor. It is a problem keeping oneself [the mother] clean after delivery. No bathing”.^39^ (Uganda) “… and then like my Mum went home and it was just like ‘oh my God I’m here on my own … It was just really, like, creepy—I think of hospitals as where you go to, die”.^43^ (UK)	Low
Social norms	*Feelings of stigma and judgement in health facilities*: adolescent girls expressed feeling unwelcome to PNC services and judged in health facilities due to age and the stigma of adolescent pregnancy.	7 studies: Robb *et al*^42^ (Scotland); Recto and Champion^45^ (USA); Atuyambe *et al*^39^ (Uganda); Hunter *et al*^43^ (UK); Peterson *et al*^36^ (Canada); Smith *et al*^40^ (UK); Muzik *et al*^44^ (USA)	“…she [midwife] kept going round to all the other women like ‘oh she’s gorgeous! What’s her name?’ [about the baby] And then she’d come to me, and she just wouldn’t ask me a thing”.^43^ (UK) “I was warned though, if you're a young mom you might get looked down on a little bit and I think that’s what she was doing cause she knew I was young and… she thought I was stupid and… she wasn’t that bad it’s just… I don’t know. I’d just had a baby I wasn’t in the best of moods. And she just… I guess didn’t understand. I didn’t give her a hard time or anything, but it seemed to me like she was giving me one”.^36^ (Canada)	High
	*Perceptions of shame and negative stereotypes of young parents*: the combination of shame due to negative stereotypes and fear of judgement affects the self-efficacy of young parents and their willingness to access care or communicate their needs when they do access care.	8 studies: Smith and Roberts^40^(UK); Dumas *et al*^46^ (USA); Atuyambe *et al*^39^ (Uganda); Vieira *et al*^34^ (Brazil); Muzik *et al*^44^ (USA); Mngadi *et al*^38^ (Eswatini); Ross *et al*^41^ (UK); Robb *et al*^42^ (Scotland)	“I don’t care really what people think of me, but it annoys me … that people are kinda like stereotypical about us”.^40^ (UK) “Those who become pregnant while still in school fear to go for health care at the health units. They fear to get ashamed or meet their own colleagues in schools. Such girls would, before pregnancy, have been proud and calling themselves virgins, so they find that they cannot stand all that shame, so they decide to keep at home”.^39^ (Uganda)	High
Experiences of care	*Acknowledgement and empathetic communication by staff at health facilities*: adolescents felt providers’ delivery of PNC was focused on checklists of tasks rather than responding to their needs or requests and offering care. Providers were often rushing to move through the items required for newborn care, leaving adolescent girls feeling unheard and without guidance. Lack of acknowledgement and empathetic communication between overwhelmed adolescent girls and busy health providers made experiences in PNC less helpful.	7 studies: Recto and Champion^45^ (USA); Peterson *et al*^36^ (Canada); Hunter *et al*^43^ (UK); Atuyambe *et al*^39^ (Uganda); Erfina *et al*^37^ (Indonesia); Muzik *et al* 44 (USA); Dumas *et al*^46^ (USA)	“They just do everything so fast. They don’t speak with you. They don’t communicate with you. They don’t even want to open up a conversation. They’re just like, ‘Oh, well you came here for this and that. Okay, next’”.^45^ (USA) “I was not taught about way to breastfeed. [they] just checked my breast milk is in. They say that I have a lot of milk ….my baby was taken by the nurse for bathing, and I didn’t see my baby being bathed”.^37^ (Indonesia)	Moderate
	*Respect and trust-building in care*: where health providers took a calm and patient approach, adolescents felt more respected and comfortable enough to ask questions and voice concerns. Trust was built through offering social support, care and providing tailored information on what to expect following birth. Trust was eroded when adolescent girls felt unheard, neglected, disrespected or handled roughly during the continuum of delivery to PNC.	10 studies: Recto and Champion^45^ (USA) Peterson *et al*^36^ (Canada); Hunter *et al*^43^ (UK); Atuyambe *et al*^39^ (Uganda); Erfina *et al*^37^ (Indonesia); Muzik *et al*^44^ (USA); Dumas *et al*^46^ (USA); Mngadi *et al*^38^ (Eswatini); Robb *et al*^42^ (Scotland); Ross *et al*^41^ (UK)	“Some nurses were more, I guess, calm. So, it was easier to talk to them than the nurses that were sort of rushing through every now and then… I mean when they rush through then its uh … you sort of forget the questions that you were gonna ask in a way”.^36^ (Canada) “I was just left, and then when I was gonna like be moved up onto the ward the nurse come and she just like sort of grabbed [baby] and tried to like ram her on to my breast and that”.^43^ (UK)	High
	*Instrumental and informational support on newborn care*: adolescent parents expressed anxiety around a lack of skill in or knowledge of newborn care (eg, feeding, bathing, cord cleaning) due to not receiving information nor guidance during PNC to learn newborn care skills; often, due to expectations of judgement around not being seen as capable, they feared asking questions or seeking more tailored support. Across participants, adolescent girls described different levels of support from providers even within the same settings.	12 studies: Robb *et al*^42^ (UK); Vieira *et al*^34^ (Brazil); Bergamaschi and Praça^32^ (Brazil); Dumas *et al*^46^ (USA); Erfina *et al*^37^ (Indonesia); da Silva *et al*^33^ (Brazil); de Melo *et al*^35^ (Brazil); Peterson *et al*^36^ (Canada); Muzik *et al*^44^ (USA); Mngadi *et al*^38^ (Eswatini); Hunter *et al*^43^ (UK); Ross *et al*^41^ (UK)	“… at the hospital I didn’t get any support at all, you were put to your ward and left. I didn’t get asked how I was feeding her, and I was bottle-feeding her, and I didn’t get told that I had to collect the bottles myself and put them away myself, so I was left and didn’t know what to do. I didn’t get asked if I knew how to change a nappy… I knew like my friend when my friend had her baby and that’s how I knew how to change a nappy …”^42^ (UK) “I know there would be young dads with the same worries that I have got, and I would like to find out how they are coping with it.… It would be nice to have some kind of guidance especially for first time dads or young dads as well that are kind of scared of what the future is going to be like”—young father.^41^ (UK)	High
	*Psychosocial support*: in high-income and upper middle-income settings, adolescents girls feel they lack knowledge on recognising the signs of postnatal depression and have the added pressure of fear around early parenting and feelings of isolation, emotional overwhelm or failure.	5 studies: Recto and 2018 Champion^45^ (USA); Muzik *et al*^44^ (USA); Dumas *et al*^46^ (USA); Vieira *et al*^34^ (Brazil); Hunter *et al*^43^ (UK)	“He made me feel like I wasn’t crazy. He made me accept the depression… My OB/Gyn said that it’s normal for mothers to experience depression during and after pregnancy… If they say it’s normal, I think it would make them feel better knowing they're not alone”.^45^ (USA) “No one ever says to you … it’s like normal to not be able to do it … I just felt like a complete failure, because no one, had explained to me—that I weren’t the only one”.^43^ (UK)	Low-to-moderate
	*Sexual and reproductive health information needs*: adolescent girls view sexual health and contraception as important but their access to information on this topic is limited. Adolescent girls’ preferences for sexual and reproductive health services were not always prioritised or available.	4 studies: Mngadi *et al*^38^ (Swaziland); Vieira *et al*^34^ (Brazil); Dumas *et al*^46^ (USA); Erfina *et al*^37^ (Indonesia)	“It was hectic. I didn’t get back to the doctor until the baby was 4 months old. By then I was pregnant again”.^46^ (USA) “Waiting the forty days to have sex, not to get pregnant again, not to burst what we have inside, we have to be careful. Because the doctor said that we may get depressed, get a lot of things, that it shatters everything inside”. 34 (Brazil)	Low
Tailored support needs	*Navigating parenthood responsibilities at a young age*: adolescents feel overwhelmed by the sudden transition to both adulthood and motherhood. They feel the need for PNC to include information on childcare as well as social support in managing the next stages of adulthood and the identity transition required. They express a lack of knowledge around available PNC or parenting support services	9 studies: Dumas *et al*^46^ (USA); Bergamaschi and Praça^32^ (Brazil); Muzik *et al*^44^ (USA); Atuyambe *et al*^39^ (Uganda); Mgnadi *et al*^38^ (Swaziland); Vieira *et al*^34^ (Brazil); da Silva *et al*^33^ (Brazil); Erfina *et al*^37^ (Indonesia); Ross *et al*^41^ (UK)	“We do not have jobs… have no income for survival and looking after our kids… that is why most girls have ended up becoming pregnant early or turning out as commercial sex workers in Kampala”.^39^ (Uganda) “Now I have more responsibility, I have to wake up at dawn to breastfeed, I worry about everything”.^34^ (Brazil)	Moderate
	*Peer support*: in studies from HIC settings, hearing peers experiencing similar challenges—both in terms of newborn care and parenthood during adolescence—and sharing information, through either informal and/or structured peer support, can alleviate some of the pressures and feelings of inadequacy and of being overwhelmed. Peer support groups were seen as an important source of emotional support and encouragement during the transition to parenthood.	3 studies: Hunter *et al*^43^ (UK); Muzik *et al*^44^ (USA); Ross *et al*^41^ (UK)	“I think I’d rather hear I’m doing well from somebody that done it. Quite recently as well … than… say a midwife that’s never had children”.^43^ (UK) “Yeah and it’s easier to speak up if you hear somebody else talkin’ you think maybe I shouldn’t say nothing but if somebody else say something you like, ok well, I kinda understand that I could speak on some of my situations”.^44^ (USA)	Moderate evidence only from HIC
	*Family support*: family support including practical, emotional and psychosocial support—and especially from the adolescents’ own mothers—is heavily relied on during the postnatal period; therefore, family members’ engagement, opinions and guidance on health-seeking behaviours affect uptake of PNC.	8 studies: Vieira *et al*^34^ (Brazil); Dumas *et al*^46^ (USA); Muzik *et al*^44^ (USA); de Melo *et al*^35^ (Brazil); Bergamaschi and Praça^32^ (Brazil); da Silva *et al*^33^ (Brazil); Mngadi *et al*^38^ (Eswatini); Atuyambe *et al*^39^ (Uganda)	“I had the support of all my family… They help me a lot… I take good care of the baby, I give baths, breastfeed, but there are several things that my mother has to watch me, to teach me… If my mother were not helping me, I do not know what I would have done”.^32^ (Brazil)	Moderate
	*Partner support*: the exclusion and/or lack of engagement of young partners in antenatal support also contributed to lower uptake of PNC with adolescent girls stating that partner inclusion would enhance their uptake of services. Young fathers also expressed a desire to be better informed and involved in order to more effectively support their partners.	3 studies: Ross *et al*^41^ (UK); Smith *et al*^40^ (UK); Muzik *et al*^44^ (USA)	“…they’ve never spoken to me, never”—young father-to-be”^40^ (UK) “‘midhusbands’ should be allocated to new fathers, as midwives help mothers”—focus group of young fathers^41^ (UK)	Low

GRADE, Grading of Recommendations, Assessment, Development and Evaluations; HIC, high-income country; PNC, postnatal care.

### Themes identified from included studies

#### Resources and access

Structural barriers to accessing PNC included lack of health insurance, not having accessed ANC, poor availability and cost of transportation to health facilities, and direct costs (eg, consultation fees) associated with PNC services. Lack of health insurance, transportation barriers, and costs associated with private delivery and PNC services contributed to adolescents seeking services from alternative sources including emergency rooms, traditional birth attendants or family members.^39 46^ Furthermore, a lack of information and knowledge regarding available maternal and neonatal care services and not having accessed ANC impacted PNC uptake as well as adolescent girls’ comfort and uncertainty with these services.^39 44 46^ In some cases, this uncertainty around the appropriateness of available services—and their alignment with adolescents’ preferences—played an important role in the choice of where adolescent girls went for delivery and PNC.^39^ Suggestions made by adolescent girls to improve awareness of available services included social media, door-to-door flyers, local television, and internet advertisements.^44^ The integrated Medical Home model—whereby multiple services can be found in one accessible setting, one-on-one care and home visits by midwives or doulas, and community events such as ‘Community Showers’—local events run by non-profits for social, informational and instrumental support to new mothers—were also indicated by adolescent girls and their partners as being effective means of enhancing awareness, accessibility and therefore use of PNC.^38 42 44 46^

Another factor affecting health-seeking behaviours is the physical state of health facilities and available resources. In two studies, adolescent girls explicitly referenced the physical environment—including cleanliness, infrastructure, and ambiance—when describing their perceptions and attitudes towards seeking PNC at health facilities and hospitals.^39 43^ Further high-quality evidence on adolescent girls’ experiences in accessing appropriate PNC resources and services—across different population groups and settings—is required to inform implementation and scale-up of effective PNC programmes.

#### Social norms

An important theme specific to uptake of PNC by adolescent girls is the stigma associated with adolescent pregnancy and the role of shame and judgement in whether and how adolescents seek and experience PNC. Young parents feel encumbered by the perceived judgement from others and the stereotypes of adolescent parents as being irresponsible, uneducated and lacking prospects.^40 42–46^ While some have been able to overcome these judgements, they continue to cite them as reasons why PNC may not feel welcoming to adolescent girls.^40^ Some adolescent girls and their partners also voiced the fear of being perceived as unable to care for their babies and thus being reported to social services as reasons for low uptake of PNC.^42^

Furthermore, when adolescent girls did access care, fear of being judged and stigmatised at health facilities by both health providers and other mothers affected adolescent girls’ experiences of care, including their willingness to communicate their needs. The pattern observed across these studies is one of adolescents sensing shame and feelings of discomfort at being in medicalised spaces designed for older women; these sentiments were sometimes reinforced through comments made by providers or stigma sensed through being ignored or not sufficiently heard. Conversely, where providers (including midwives, nurses and physicians) spent more time with adolescents and reassured them regarding the development of their competencies and confidence in newborn care, adolescents were less likely to feel judged and more likely to make effective use of PNC.^36 45^

#### Experiences of care

In addition to the impact of stigma and perceived judgement, adolescent girls’ experiences of care were also affected by the nature of communication with health providers, availability of practical advice, information and guidance, and appropriate psychosocial support. Across the included studies, adolescents voiced a feeling of being treated in a ‘checklist’ manner whereby health providers were quickly going through a list of postnatal tasks rather than offering individualised care by listening to their needs and concerns. The respondents expressed a reluctance to ask questions due to the rushed nature of service delivery. The most frequently mentioned reason for this was that nurses, midwives, and other health providers were focused on ensuring the health of the baby and often rushing through necessary tasks, leaving adolescent girls—who stated that they were less likely to speak up than older mothers—unsure as to how to find guidance on appropriate newborn care. Lack of acknowledgement and perceived empathetic communication between overwhelmed adolescent girls and busy health providers negatively impacted adolescent girls’ experiences of PNC. Where adolescent girls voiced satisfaction with the quality of care, it was primarily associated with a calm and patient approach by health providers, making them feel that their concerns were valid.^36 41 43 45^

Our findings also suggest that while some adolescent girls were able to establish good relationships with PNC providers and could seek the support they needed, many others either did not know that such support could exist or felt that they could not ask for fear of being ridiculed. Trust was eroded when adolescent girls felt unheard, neglected, disrespected or handled roughly during the continuum of delivery to PNC. This in turn affected trust in the health system and a breakdown in communication with health providers. In some instances, adolescents felt that providers did not care and were even annoyed; examples of verbal abuse were mentioned in at least two included studies and some adolescents described being handled in a rough manner while learning to care for their newborn.^38 39^

Experiences of care were also impacted by whether adolescent girls and their partners received the types of support they sought. In general, adolescent girls and their partners expressed a need for further informational—defined as the provision of information needed to overcome barriers^47^—and instrumental support—defined as ‘the provision of tangible goods and services’^47^—in taking care of the immediate needs of the newborn (eg, bathing, feeding, cord care). Many stated that health providers were not sufficiently teaching them the skills required for newborn care nor engaging them in practicing these skills. This gap in informational and instrumental support exacerbated fear and anxiety for some and undermined the perceived effectiveness of PNC.

Where adolescent girls expressed positive experiences of PNC, it was often due to proactive support, praise and open communication from health providers that created the space for questions and voicing of concerns. Importantly, adolescents across several studies expressed the importance of a health provider informing them/acknowledging the signs of postpartum depression and reassuring them that the feelings of inadequacy and exhaustion they experienced were normal and could be addressed with mental health support. This form of psychosocial support—where articulated—was viewed as enhancing adolescent girls’ PNC experience and access to other types of supportive services. Throughout studies, adolescent girls expressed appreciation for the informational support they received during PNC that allowed them to access helpful services beyond the postnatal period. Adolescents also mentioned needing better information on sexual health and contraception during the postnatal period with some studies highlighting the dissatisfaction with available family planning resources.^37 38^ In some instances, adolescent girls found themselves pregnant again soon after giving birth and shared that access to improved and stable postpartum support as well as addressing structural barriers—such as cost, stigma, or knowledge—in accessing preferred contraceptive methods would make family planning more effective.^37 38 46^

#### Tailored support needs

Adolescents frequently spoke of feeling overwhelmed, exhausted, and ill-prepared to take on the transition to parenthood. They often wanted more support from health and social services to continue their education and professional trajectories while ensuring the well-being of their baby. Adolescent girls described the existing scope of PNC as not wide enough to understand or meet their needs, making it less appealing as a service. For example, in one study, participants were disappointed with the focus on physical examinations and contraceptive counselling.^37^ In general, adolescent girls and their partners noted that they needed more information and resources tailored to adolescent parents in the postnatal period to be able to care for their newborns and to continue their personal development. Further high-quality evidence on adolescent girls’ support needs, in particular evidence on the effectiveness of support interventions, is required to strengthen these findings.

Partners of adolescent girls specifically expressed needing more informational support and a desire for having go-to avenues to seek information that would allow them to better support their partners during the postnatal period.^41^ Where consulted, partners expressed a desire to be more involved and helpful but found that existing PNC services neither engaged nor leveraged their involvement in supporting adolescent mothers in the postnatal period.^40 41^ Adolescent girls also spoke of having partners’ support and their engagement in PNC as contributing to increased PNC uptake^40 46^; however, it should be noted that the experience of having partners who wanted to be involved and supportive was not universal across participants in the included studies.^39^

The role of emotional support from social networks during the postnatal period for adolescent girls is significant. Peer perspectives and family support are heavily relied upon according to the adolescents interviewed across studies. Peer groups, comprised of fellow adolescent girls and/or their partners, are seen as valuable in addressing some of the feelings of loneliness and anxiety. In some instances, a preference for peer group-based PNC over individual care was expressed. The solace sought in peer experiences is reflective of feeling out of place in traditional PNC due to age and stereotypes around adolescent pregnancy.

In addition to the emotional support and encouragement afforded by peer groups, some adolescents seek informational and instrumental support from family members. Family members, especially mothers of adolescents, are often one of the primary sources of consultation to support with postnatal tasks and care of the baby.

## Discussion

Uptake and perceptions of PNC among adolescent girls is affected by multiple factors including resources and access, social norms, experiences of care, and tailored support needs. Adolescent girls identified barriers to accessing PNC such as financial constraints to access, lack of information on available services, feeling unwelcome due to perceptions of stigma and shame around adolescent pregnancy, not receiving the necessary instrumental or psychosocial support, not feeling acknowledged nor heard, and not having their specific needs and preferences met.

While some access barriers for adolescent girls were similar to those expressed by postpartum women of all ages in the primary review^24^—such as financial and transportation challenges—others, in particular regarding experiences of care and social norms, presented unique challenges to adolescents.^20 39 45 46^ Adolescent girls expressed a desire for and appreciation of empathy and responsiveness of health providers to their unique needs, with many describing feeling shame in asking questions, feeling incapable, feeling that they are entering spaces not designed for them and feeling like an item on a staff member’s checklist. These point to a gap in adolescent-sensitive maternal healthcare as part of high-quality integrated PNC, as well as ANC and preconception care. The importance of addressing this gap is also noted in reviews of quantitative evidence focused on adolescent girls, where the positive impact of adolescent-focused services and continuity of care is noted to improve retention in maternal health services including ANC, delivery and PNC.^48^

Ensuring that adolescent-sensitive services exist starting from the antenatal stage and offering continuity of care by trusted providers through to the postnatal period can enhance uptake of maternal health services at every stage.^49^ Across the qualitative studies included in this review, participants often described access barriers to ANC as also impeding access to PNC, highlighting the significance of the continuum of care from ANC to delivery to PNC. Indeed, access to ANC and to delivery services often predicts access to PNC as identified by quantitative evidence around use and access to maternal healthcare among adolescent mothers in low-income and middle-income countries (LMICs).^20 49–51^

In supporting continuity of care, trust-building and good communication are essential. In this review, instances of rough handling of mothers and verbal abuse in the continuum of delivery to PNC were articulated by adolescent girls, indicating a link to disrespect and abuse previously identified in the literature on childbirth experiences across mothers of all ages.^52 53^ Where adolescent girls noted positive examples of trust, effective communication and good experiences of care, they highlighted the calmness and attentiveness of providers as well as one-on-one time and home visits as being helpful.^36 41 45^ These findings parallel findings in the primary review, demonstrating that relational support and continuity of care from health providers are viewed as essential to the uptake and acceptance of PNC interventions and are impeded when interactions with health providers are short and constrained by health workforce limitations.^24 37 43^ This points to the importance of ensuring that the health workforce also has the support and sufficient resources to meet the needs of the populations they serve as opposed to feeling pressured due to staffing shortages, poor investment in health systems and high rates of turnover and burnout.^54–57^ Addressing these health system challenges can also enhance continuity of care, including through midwife-led models highlighted in the WHO’s PNC guidelines, thereby contributing to improved trust and communication.^5 55–58^

In addition, similar to findings in other studies of maternal health service utilisation by adolescent girls, experiences of care were negatively impacted by instances of disrespect, abuse, perceived intolerance and impatience, highlighting the need for more concerted efforts in embedding empathetic approaches in the training and practice of maternal and child healthcare for all demographic groups.^53 59 60^ A review of how the experiences of women and girls influence uptake of PNC in African countries similarly highlighted trust as being built through respectful care, defined as health providers being ‘kind, supportive and attentive to women’s needs’ and disrespectful care as including ‘verbal and/or physical abuse and power imbalances between women and healthcare providers’.^61^ Building trust and respectful communication with health providers is critically important in this subpopulation, particularly due to the oftentimes higher risk of disrespectful maternity care among adolescent girls.^61^ While findings in our primary review also note mothers’ feelings of disrespect and being overlooked, the subpopulation studied in this review strongly emphasise perceived judgement, disrespect and lack of belonging in PNC, citing stigma and negative stereotypes as potential reasons.^24^

The impact of social norms—particularly attitudes towards adolescent pregnancy and childbearing, stigma, the associated shame, fear, and perceived loss of other opportunities such as access to education—are pronounced among this subpopulation. These negatively impact PNC uptake by adolescent girls due to perceptions of being judged by health providers and other mothers. This perception also impedes the willingness of young parents to voice their questions and concerns, making care less effective. Adolescent mothers are in a unique situation whereby they are balancing motherhood with the existing challenges of adolescence and transition to adulthood. Services that ignore these realities or perpetuate the feelings of shame serve as barriers to the uptake of PNC and place adolescent girls at risk of feeling alienated from available health services, with implications for physical and mental health outcomes.

This review also identified an emphasis on instrumental, informational, and psychosocial support needs among adolescent girls and their partners. The fear of not being capable of caring for their newborns and not having sufficient information on what to do was frequently cited across studies. Beyond support from providers, adolescent girls also rely on partners, family, and peers. In this review, adolescent girls noted that involvement of their partners would enhance ANC and PNC uptake. This finding is supported by quantitative evidence as well where adolescent girls accompanied by a partner during ANC have higher rates of PNC utilisation.^49^ In addition, adolescent girls’ partners expressed a desire to be more actively engaged and to have better access to information to effectively support their partners. Approaches that seek to increase partner engagement should account for potential unintended consequences, such as the risk of further stigmatising those without partners. Further discussion of the perceptions of partners and families and different levels of engagement in PNC is included in a companion review by Finlayson *et al* stemming from the same source QES.

The influence of peers and family members can also play a critical role in the uptake of different aspects of PNC as articulated in a review of factors influencing breast feeding among adolescents.^62^ This has important implications for how health and social services can involve family members in enhancing PNC uptake. Highlighting the instrumental, emotional, and psychosocial support needs of adolescents and their experiences in receiving these from healthcare providers, partners, family members, and peers is important in designing interventions that appropriately leverage support networks. Meeting the tailored informational and support needs of adolescents is critical in counteracting the disempowerment that adolescent girls and their partners may feel. Counteracting this disempowerment may require adapting approaches used to support the transition of adolescents from paediatric services. These approaches include person-centred care, family partnered care, developmentally appropriate care, and coordination of specialist and community-based care services.^63^ Furthermore, a lack of information and fear around how to appropriately care and provide for a newborn exacerbates feelings of anxiety around the transition to motherhood since adolescents may not be aware that their postpartum challenges are shared by others and that there are resources to help support them. Expanding family partnered care to facilitate peer-supported empowerment and community building can address some of these challenges. As indicated by positive experiences shared by adolescents, interventions to enhance trust and respectful care, adolescent-sensitive services, and meaningful adolescent and youth engagement can be effective means of enhancing PNC uptake. Examples of helpful programmes identified by adolescents in this review include ‘Community Showers’ that contribute to enhancing informational and social support by bringing together support networks and integrated ‘Medical Home’ models of care that remove access barriers by merging together different sources of instrumental and psychosocial support.^44 46^ In the design, implementation and evaluation of programmes and interventions like these, particular attention should be paid to ensuring cultural safety^64^ and mitigating disparities by racialised group, urban residence, socioeconomic status, and other structural determinants of health inequites.^9^

### Limitations and strengths

This analysis highlights the specific needs of an understudied subpopulation. By analysing a focused subset of studies from a larger, rigorous QES with no geographic or linguistic restrictions, we have highlighted the reported needs of adolescent girls, their newborns, and their partners in the postnatal period and identified unique challenges and concerns expressed by this group. Addressing these can serve to make high-quality integrated PNC more inclusive and effective.

The review is limited to the topics, questions, geographic areas and demographic groups explored in the included papers and therefore reflects the biases inherent in what is present as well as what is absent in the current literature on the views of adolescent girls on routine PNC. For example, despite the global scope of the source review, there is a lack of geographic and subregional diversity in this review with lower representation from LMICs, limiting the generalisability of results. A recent review of women’s experiences and perceptions of PNC across sub-Saharan Africa highlights many similar themes, particularly poor experiences of care, physical access barriers, psychosocial challenges and stigma and shame among vulnerable women—with adolescents identified as a vulnerable group.^61^ However, as evidenced by the small number of studies included in this study, a focus on adolescent girls’ experiences with, perceptions of, and needs regarding PNC remains sparse. Furthermore, although the search was not limited to facility-based studies, threats to representativeness of the sample include a skew towards facility-based recruitment which contributes to less diversity in terms of urban residence and community and family structure as well as a limited ability to draw comparisons between facility-based and home and community-based maternal and neonatal care. Additionally, adolescent-specific programmes and initiatives that do not explicitly use terminology around PNC may have not been included in the review resulting in fewer examples of adolescent-specific programming.

It is also important to note that while WHO defines adolescents as ages 10–19 years, the term ‘young people’ used in some of the included studies, covers the age ranges 10–24 years. The wider age range may have implications for which factors are viewed as important in PNC uptake and whether these are applicable across all included ages. Many of the review findings would benefit from further data (ie, more studies across different populations) to increase the level of confidence in the thematic finding. It is also worth noting that by virtue of targeting this subpopulation, the studies included in this review were likely to focus data collection and analysis on challenges viewed as specific to adolescents. This may present a bias in the themes that are highlighted by, for example, emphasising the feelings of stigma and shame and presenting less information with regard to adolescent girls’ feelings and perceptions around challenges or enablers that would also apply to adult women since these were asked about less frequently. Furthermore, most studies focus on ANC or labour and childbirth, indicating a paucity of evidence for PNC.

Finally, there is a lack of intersectional analyses of equitable access to high-quality PNC for adolescents across different demographics (eg, urban residence, socioeconomic status, sexual and gender minority group, and racialised group). A more dedicated focus in future research on the postnatal period and its impact on adolescent girls and their newborns across different geographic areas and subpopulation groups would help address these issues.

## Conclusion

PNC is an effective means of reducing maternal and newborn morbidity and mortality. While some of the barriers to the uptake of routine PNC expressed by adolescent girls are similar to those identified by women of all ages, a focused QES on this subpopulation reveals unique challenges for adolescent girls and their partners. Key themes highlight concerns around stigma and a desire for empathy, support, and guidance from health providers during the PNC period to facilitate the transition and negotiation between adolescence and parenthood. These themes point to the need for investment in adolescent-sensitive care through improvements in continuity of care, targeted in-service and preservice training for health providers as well as ongoing supportive supervision, support for family partnered care, diversified platforms of access to PNC—including community-based programmes—and establishment of peer support networks. Furthermore, this review demonstrates the importance of investment in qualitative evidence to capture voices that are under-represented and to inform policies and programmes that are more responsive to the needs and lived realities of diverse populations.

## Data Availability

No data are available.
