# Supplementary material for: Factors that influence the uptake of postnatal care among adolescent girls: a qualitative evidence synthesis
Source: BMJ Glob Health. 2023 May 3;8(Suppl 2):e011560. doi: 10.1136/bmjgh-2022-011560 (PMC10163540; doi:10.1136/bmjgh-2022-011560)
Supplement: Supplementary data [file bmjgh-2022-011560supp001.pdf]

Appendix 1. Data Extraction Form

| POSTNATAL CARE REVIEW QES - DATA EXTRACTION | |
|---|---|
| **Reviewer** | |
| **Date** | |
| **1. IDENTIFICATION** | |
| **ID #** | |
| **Author** | |
| **Year** | |
| **Title** | |
| **2. SETTING & DEMOGRAPHICS** | |
| **Focus of the article (maternal, neonatal, both)** | |
| **Location of study (city, district, state, country)** | |
| **Urban or rural?** | |
| **Resource setting (HIC, LMIC, LIC)** | |
| **Where based? (Facility, community, home, etc)** | |
| **Language of article** | |
| **# of participants (total; specify how many per FGD/how many interviewed)** | |
| **Who are the participants/respondents?** | |
| **Details about participant characteristics** | |
| **3. TIMING OF STUDY** | |
| **Time period of focus (pregnancy, intrapartum postpartum)** | |
| **Time period of PNC contact addressed: i) within 24 hours of the birth, ii) at least on day three after birth, ii) in the second week after** | |

| | |
|---|---|
| **birth between 7 and 14 days; iv) at six weeks after birth; v) other** | |
| **4. LITERATURE REVIEW** | |
| **Literature review undertaken/referred to (Y/N)** | |
| **Focus of review appropriate and comprehensive (Y/N)** | |
| **5. STUDY DESIGN & DATA COLLECTION** | |
| **a.i) Aims/objectives (list)** | |
| **a.ii) Aims/objectives appropriate? (Y/N)** | |
| **b.i) Study design (log), i.e. descriptive, grounded theory, phenomenology, etc;** | |
| **b.ii) Study design appropriate (Y/N)** | |
| **c) Sample size appropriate y/n (clearly explained?)** | |
| **d) Reflexivity present? y/n (Concerns about bias? -indicate if own concerns or author's concerns)** | |
| **e) What data collection methods were used? (i.e focus group, in-depth interview, survey, etc;)** | |
| **f) Data collection methods appropriate y/n (described and explained - including participant recruitment)** | |
| **g) Other information about study design?** | |
| **6. ETHICS** | |
| **Ethical issues addressed y/n (explain if necessary)** | |
| **7. DATA ANALYSIS** | |

| | |
|---|---|
| **Data analysis method appropriate y/n (clearly explained)?** | |
| **8. FINDINGS** | |
| **Do the findings reflect the data? y/n (Clear themes supported by quotes)** | |
| **Are the findings relevant? y/n (generalizability, transferability, limitations all addressed)** | |
| **QUALITY APPRAISAL SCORE (A,B,C,D*) based on 3-8 above. [*D = poor quality: Exclude]** | |

**9. QUALITATIVE DATA - KEY THEMES ***insert more rows as necessary**

| **Theme (author interpretation)** | Author's interpretation (i.e.: author text) ****include page numbers*** | Participant quotation ****include page numbers*** | What level does this theme refer to? (individual, health system, etc.) | If provider mentioned, specify: physician, nurse, midwife, CHW, other staff | Special focus population? (Migrant/Refugee, Comorbidities, Adolescent, etc.) | **THEME: Resources and Access; Behaviours and attitudes; External Influences; What women want and need; Other** | Reviewer comments |
|---|---|---|---|---|---|---|---|
| | | | | | | | |
